# Identification and verification of CCNB1 as a potential prognostic biomarker by comprehensive analysis

**DOI:** 10.1038/s41598-022-20615-8

**Published:** 2022-09-27

**Authors:** Yinzhen Zeng, Rong Fan

**Affiliations:** 1 Department of Anesthesiology, Nanfang Hospital of Southern Medical University, Guangzhou, 510515 Guangdong People’s Republic of China; 2 Central Laboratory, Tianjin Xiqing Hospital, Xiqing Road 403rd, Tianjin, 300380 People’s Republic of China

**Keywords:** Gastroenterology, Cancer, Gastrointestinal diseases, Computational biology and bioinformatics, Data mining, Functional clustering, Microarrays, Cancer, Gastrointestinal cancer, Tumour biomarkers

## Abstract

As one of the most common types of pancreatic cancer, pancreatic ductal adenocarcinoma (PDAC) is highly invasive and lethal. This study aims to develop biomarkers and targets for the diagnosis and treatment of PDAC. Differentially expressed genes (DEGs) were screened via GEO2R, protein network was constructed through STRING and Cytoscape. Functional enrichment analysis was performed, followed by survival analysis and expression validation. A total of 115 DEGs were identified, including 108 upregulated and 7 downregulated genes. After enrichment, survival analysis, one potential gene, Cyclin B1 (CCNB1), was selected for further expression verification at the mRNA and protein level. Taker together, CCNB1 may act as a potential biomarker which provided new idea for elucidation of the pathogenesis of PDAC.

## Introduction

More than 200,000 people die from pancreatic cancer each year, one of the highly malignant tumors of the digestive system, which makes pancreatic cancer the seventh leading cause of cancer death worldwide^1^. Pancreatic cancer has nearly the same number of deaths and cases, with the highest rates in Europe and North America^2^. Among all cancer types, pancreatic cancer has the lowest 5-year survival rate of 3%-15%, and is projected to be the second leading cause of cancer-related death in the United States by 2030^3,4^. PDAC accounts for over 90 percent of pancreatic malignancies, and it is one of the most prevalent cancer type^5,6^. Compared with lung, breast, colorectal and gastric cancers, PDAC has a lower incidence but higher mortality. The clinical prognosis of PDAC is generally poor, with one-year and five-year survival ratio of only 24% and 9%, respectively^7,8^. Genetics, smoking, high-fat diet and chronic pancreatitis, etc. are closely related to the occurrence of PDAC^9^. At present, surgical resection remains the preferred options for the treatment of PDAC. However, due to the latent and occult nature of pancreatic cancer, most patients are diagnosed too late, and the tumor tissue has already invaded and formed distant metastasis, which reduced the effect of surgical treatment^10^. Furthermore, postoperative chemotherapy is unsatisfactory due to drug resistance^11^. Therefore, finding specific markers for early diagnosis and treatment is of great significance for improving the prognosis and survival ratio of PDAC patients.

Over the past few years, high-throughput sequencing and gene chip technology are applied in many fields of biology and medicine, such as the discovery of gene variants and methylation modifications that are closely related to tumor progression, which help to classify tumors according to histology and clinical data and identify cancer-related genes and biological pathways^12–14^. In recent years, bioinformatics, an emerging discipline that integrates mathematics and biology, makes large-scale microarray data analysis more convenient and effective by obtaining gene expression profile information. It is an effective method to systematically screen tumor-related genes. It is very helpful to explore the relevant molecular mechanisms^15–17^.

In this study, the differently expressed genes (DEGs) between normal and PDAC tissues were selected out of the Gene Expression Omnibus (GEO) database. After Gene Ontology (GO) functional enrichment and expression validation, four genes were considered as potential biomarkers for survival prognostics of patients with pancreatic cancer.

## Results

### Identification of DEGs in pancreatic carcinoma

**Figure 1:**
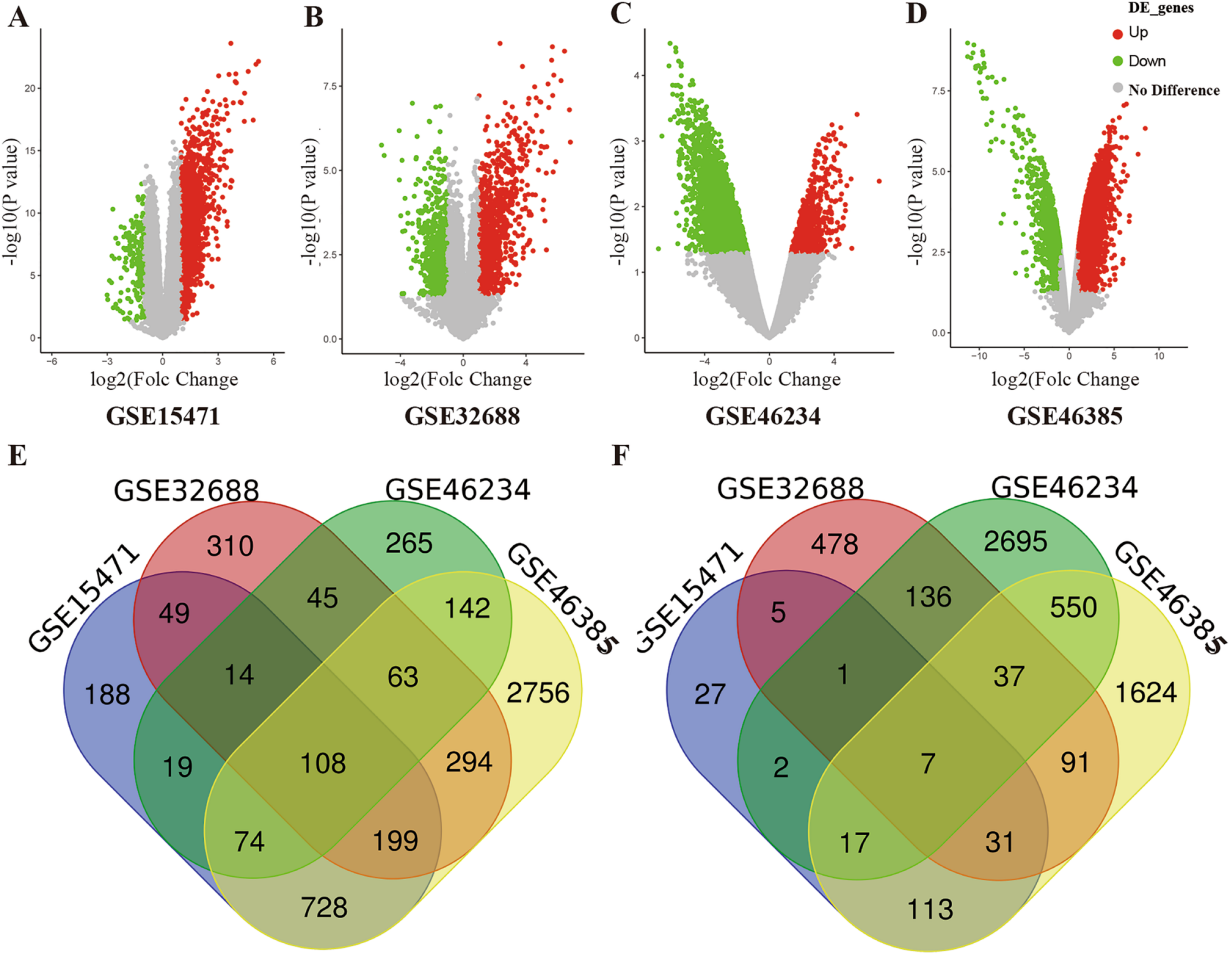
Selection of 115 common DEGs from three datasets (GSE15471, GSE32688, GSE46234 and GSE46385) (**A–D**). Volcano plot of DEGs from the three datasets; (**E**) 108 DEGs are up-regulated (logFC ≥ 1); (**F**) 7 DEGs are down-regulated (logFC ≤ −1).

In the present study, we selected three GEO datasets which covered 65 pancreatic ductal adenocarcinoma tissues and 50 normal pancreatic tissues. By using |logFC|≥ 1 and *P* < 0.05 as cut-off criterion, we obtained 1379, 1082, 730, 4634 upregulated genes and 203, 786, 3445, 2470 downregulated genes in GSE15471, 32688, 46234 and 46385, respectively. We found 115 common DEGs in the PDAC samples, including 7 down-regulated genes and 108 up-regulated genes (Fig. 1 and Table 1) using the Venn diagram software.

**Table 1:** 115 commonly DEGs were screened from four profile datasets, including 108 upregulated and 7 downregulated genes in PDAC tissues compared to normal tissues.

DEGs	Genes symbol
Upregulated	CCNB1, COL1A1, MIR6787///SLC16A3, CDK1, PAQR8, OAS1, SDR16C5, MELK, MUC20, GPX2, HN1, ITGA2, MALL, ANXA2, CEACAM5, MELTF, LGALS3, TMPRSS3, TMC5, C11orf80, TNFRSF21, CDC42EP5, JUP, MARCKSL1, CENPK, IGFBP3, COL1A2, AGR2, ST6GALNAC1, SLC44A4, BAIAP2L1, C1orf106, CD55, ITGB4, DCBLD2, FZD2, SLC12A2, EFNA1, LGALS3BP, CAPG, ADAM9, GPRC5A, KCNK1, SFN, ACSL5, ISG15, COL3A1, EPPK1, LOC400043, IFI6, LY75, NQO1, SDC1, TOP2A, RTP4, S100A10, DOCK5, EGLN3, LCN2, COL5A1, CTHRC1, AHNAK2, MTMR11, C15orf48, CEACAM6, PCDH7, PERP, LAMB3, AOC1, OSBPL3, PI3, PTTG1, POSTN, CTSE, NT5DC2, GPX8, KRT19, MX1, LAMC2, GCNT3, CEACAM1, TFF1, PYCARD, S100P, BIK, CLDN4, ELF3, DUOX2, OAS3, SDC4, DDX60, SLPI, ADAM10, S100A6, S100A11, ADGRG6, TMEM45B, ASPM, INHBA, IFIT1, HK2, ERO1A, C19orf33, TSPAN1, FERMT1, PMEPA1, FXYD3, MAL2
Downregulated	BTG2, IAPP, ERO1B, DMD, ALB, PDK4, TEX11

### Functional enrichment analysis of the DEGs

**Figure 2:**
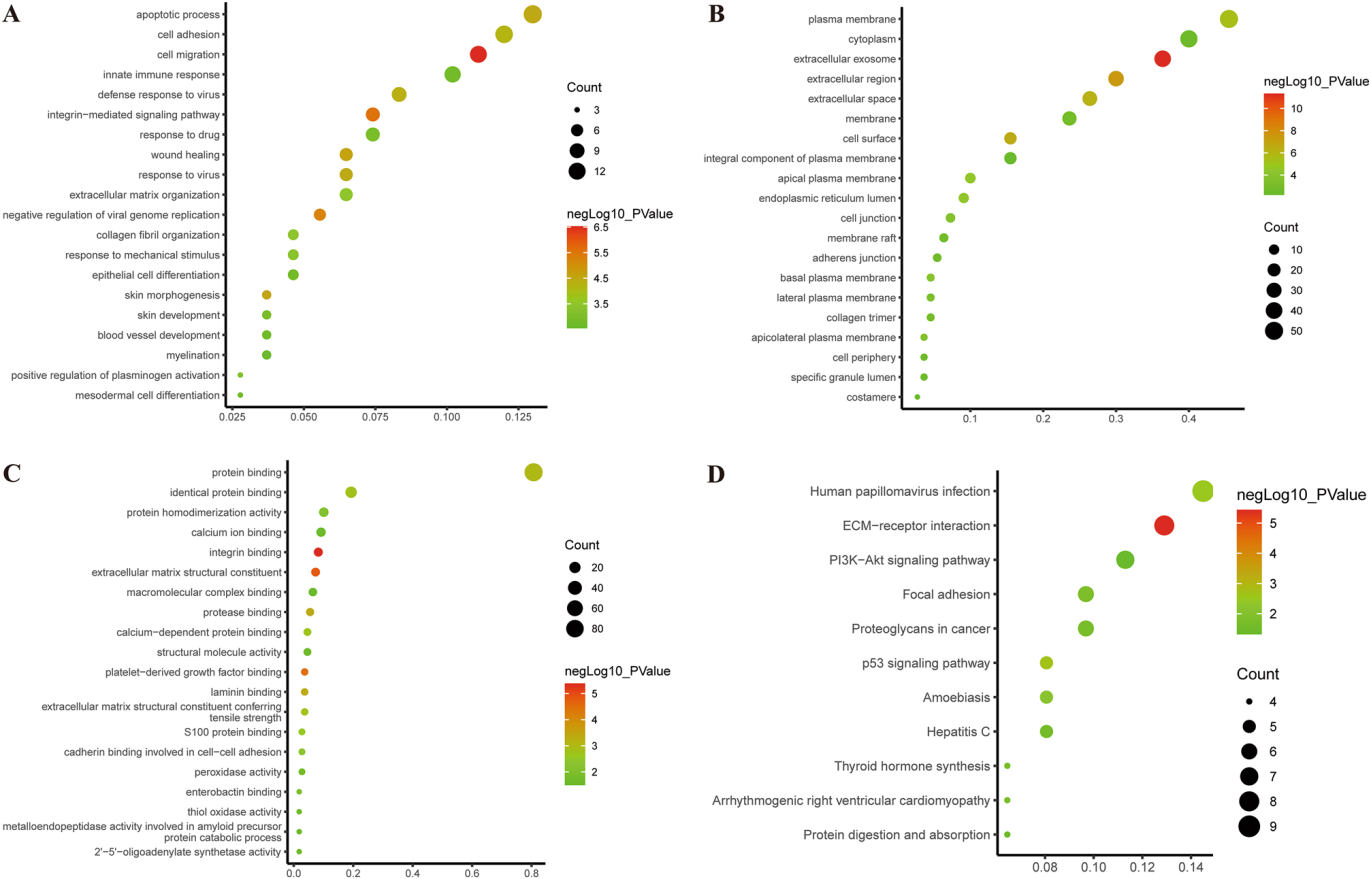
The top 20 GO and significantly enriched KEGG pathways. (**A**) BP; (**B**) CC; (**C**) MF; (**D**) KEGG pathways. The Y-axis indicates remarkably enriched items, the X-axis shows the degree of enrichment; P-value are indicated by the color of the dots, and the size of the dots represents the number genes enriched in the GO and KEGG pathways.

In order to gain insight into the functional properties of DEGs, we performed gene functional analysis via DAVID, and identified 115 significant enrichment categories, including BP (57), CC (37), MF (21). As shown in Fig. 2 and Supplementary Table 1–3, the DEGs were mostly clustered in extracellular matrix organization, cell adhesion and cell migration in terms of BP. With regard to CC, the DEGSs were particularly enriched in extracellular matrix, extracellular region, extracellular space and focal adhesion. As for the MF group, the DEGs were strongly enriched in protein binding, integrin binding, laminin binding and extracellular matrix structural constituent. Pathway analysis uncovered that the DEGs were enriched in 11 pathways including ECM-receptor interaction, PI3K/Akt signaling pathway, Focal adhesion and p53 signaling pathway (Fig. 2D and Supplementary Table 4).

### PPI network establishment and module analysis

**Figure 3:**
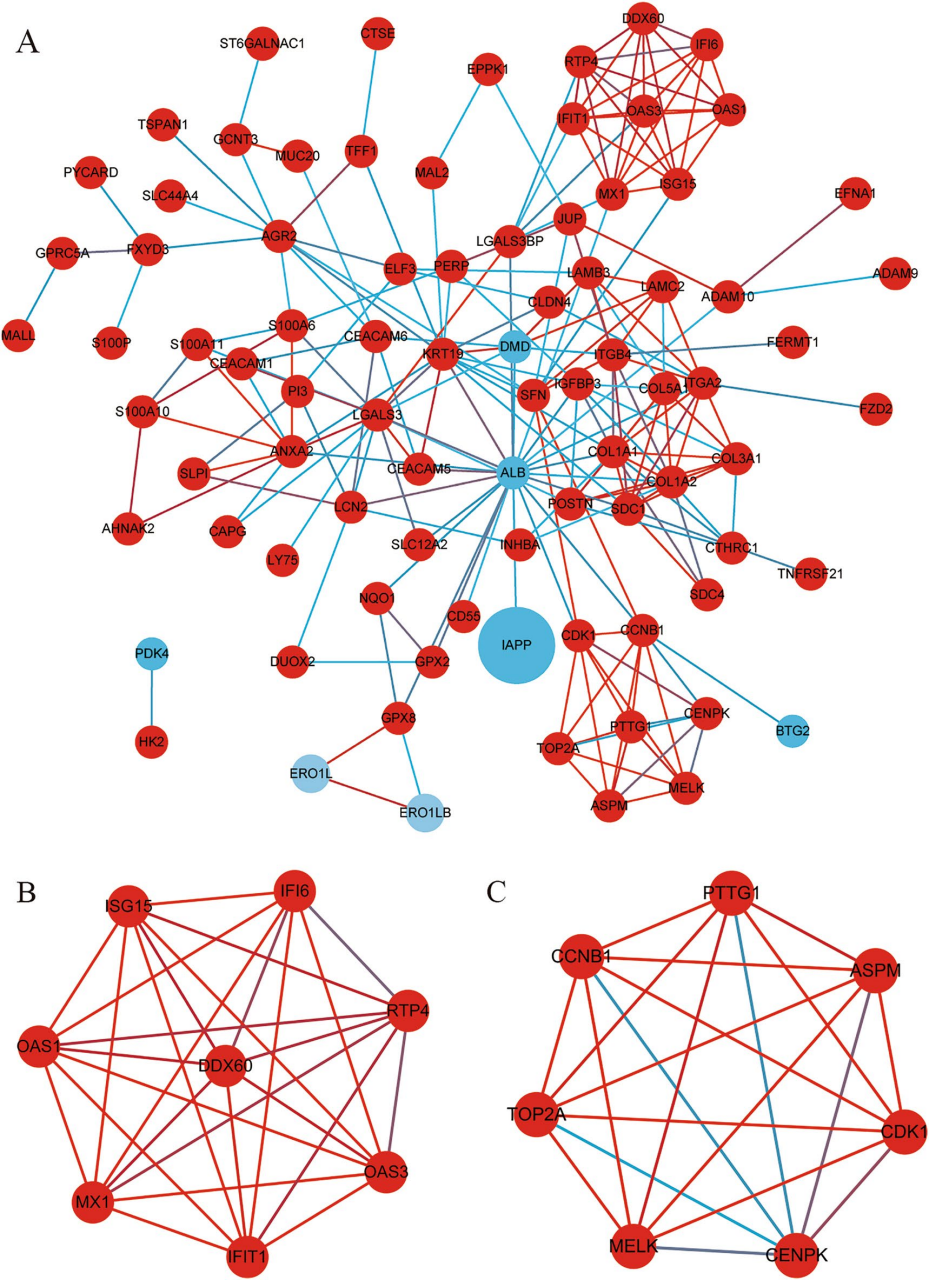
PPI network constructed and module analysis using STRING and Cytoscape. (**A**) PPI network; (**B**) Module 1; (**C**) Module 2. Every node represents a protein; edges represent protein interactions; red circles indicate up-regulated DEGs, while blue ones indicate down-regulated DEGs.

Based on the STRING database, we visualized the PPI network of DEGs by cytoscape software which was constructed with 113 nodes and 212 edges, including 108 up-regulated and 7 down-regulated genes. Furthermore, we conducted cluster analysis, and obtained 15 central nodes which are all up-regulated (Fig. 3 and Table 2).

**Table 2:** 15 central genes were selected by STRING and Cytoscape software from PPI network.

DEGs	Gene symbol
Upregulated	ISG15, IFIT1, MX1, IFI6, RTP4, OAS3, DDX60, OAS1, ASPM, TOP2A, PTTG1, CENPK, CDK1, CCNB1, MELK

### Selection of hub genes and validation of the expression levels

**Figure 4:**
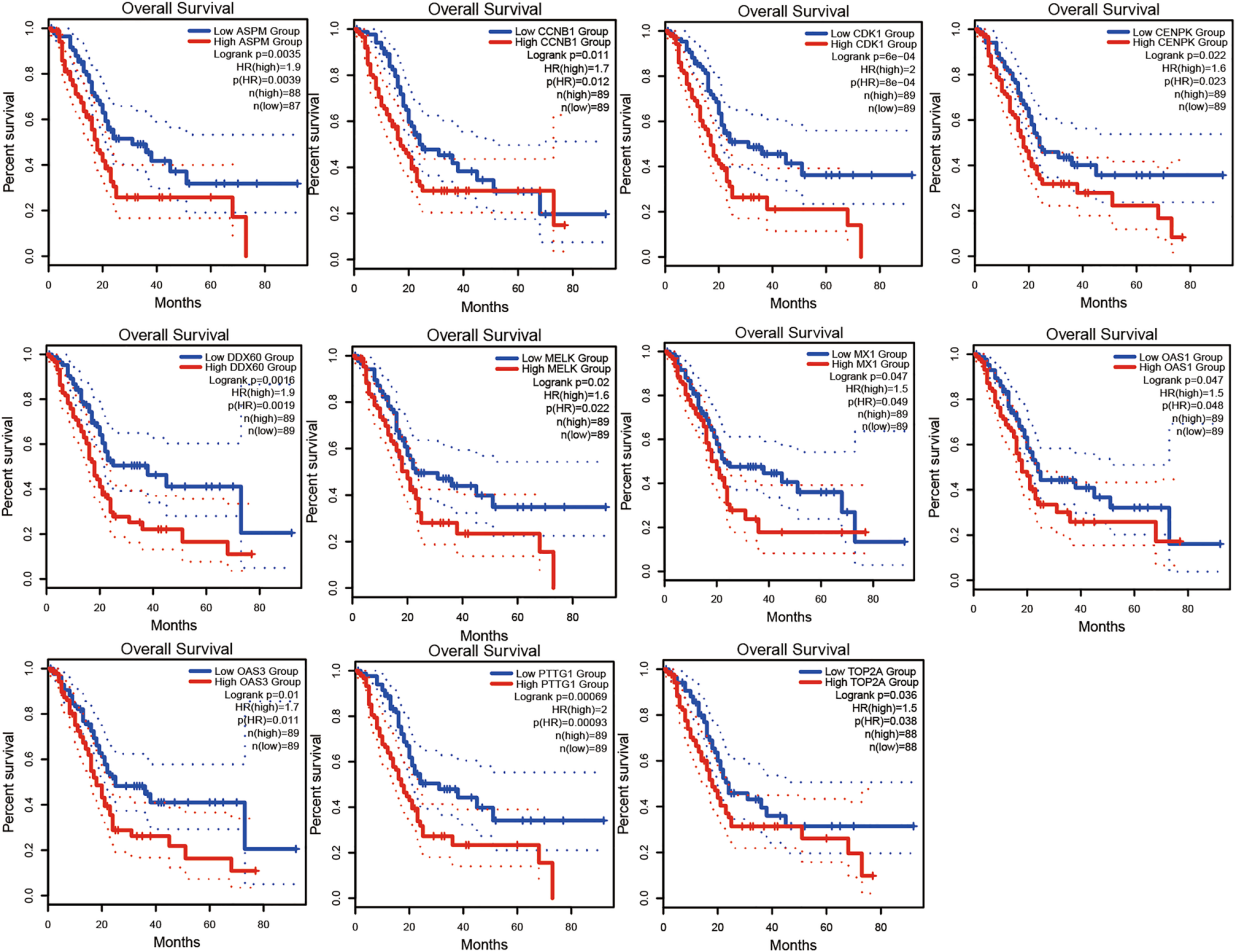
Analysis of correlation between the expression of central genes and the overall survival of PDAC patients via GEPIA.

**Figure 5:**
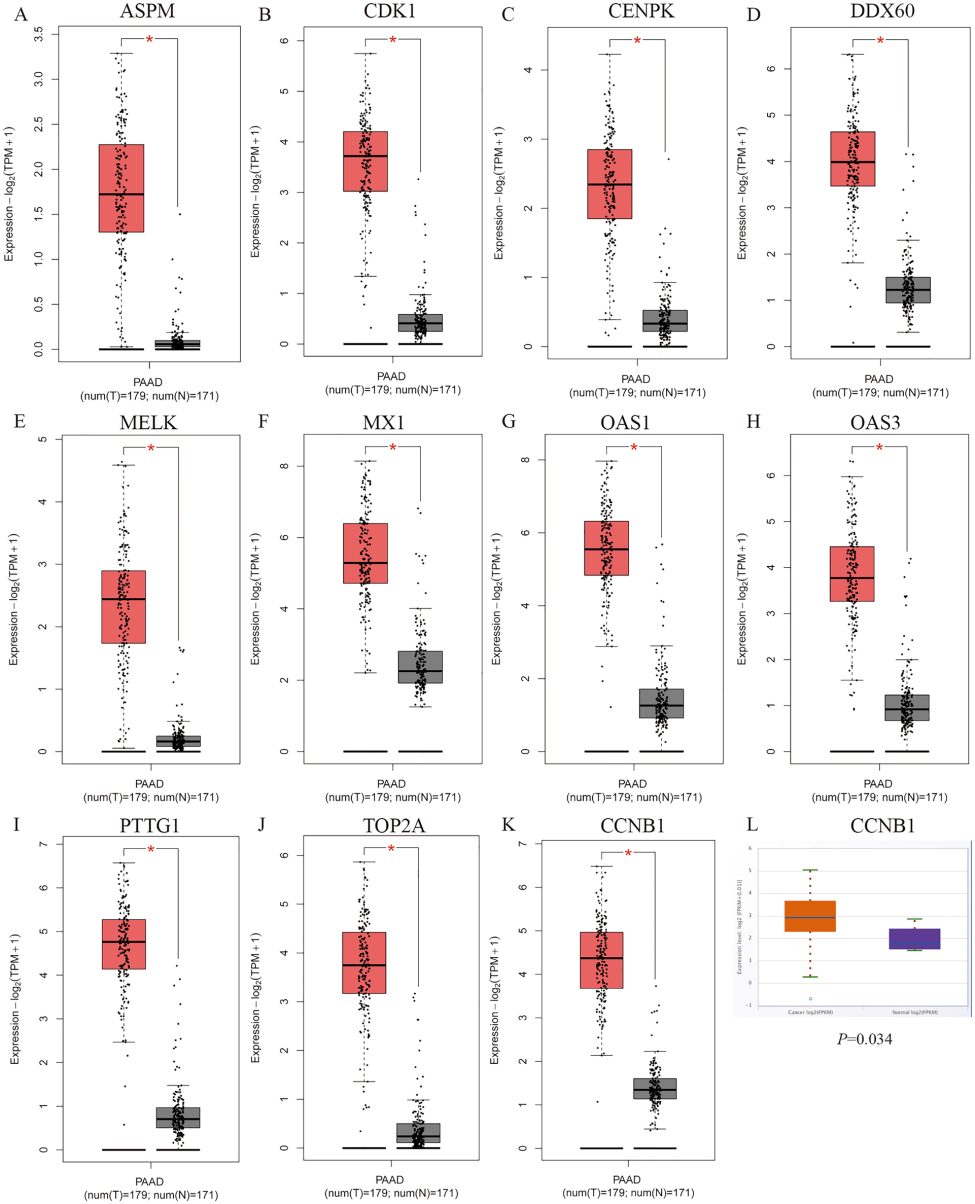
Expression analysis of central genes (**A–K**). Expression analysis via GEPIA; (**L**) expression analysis via ENCORI.

**Figure 6:**
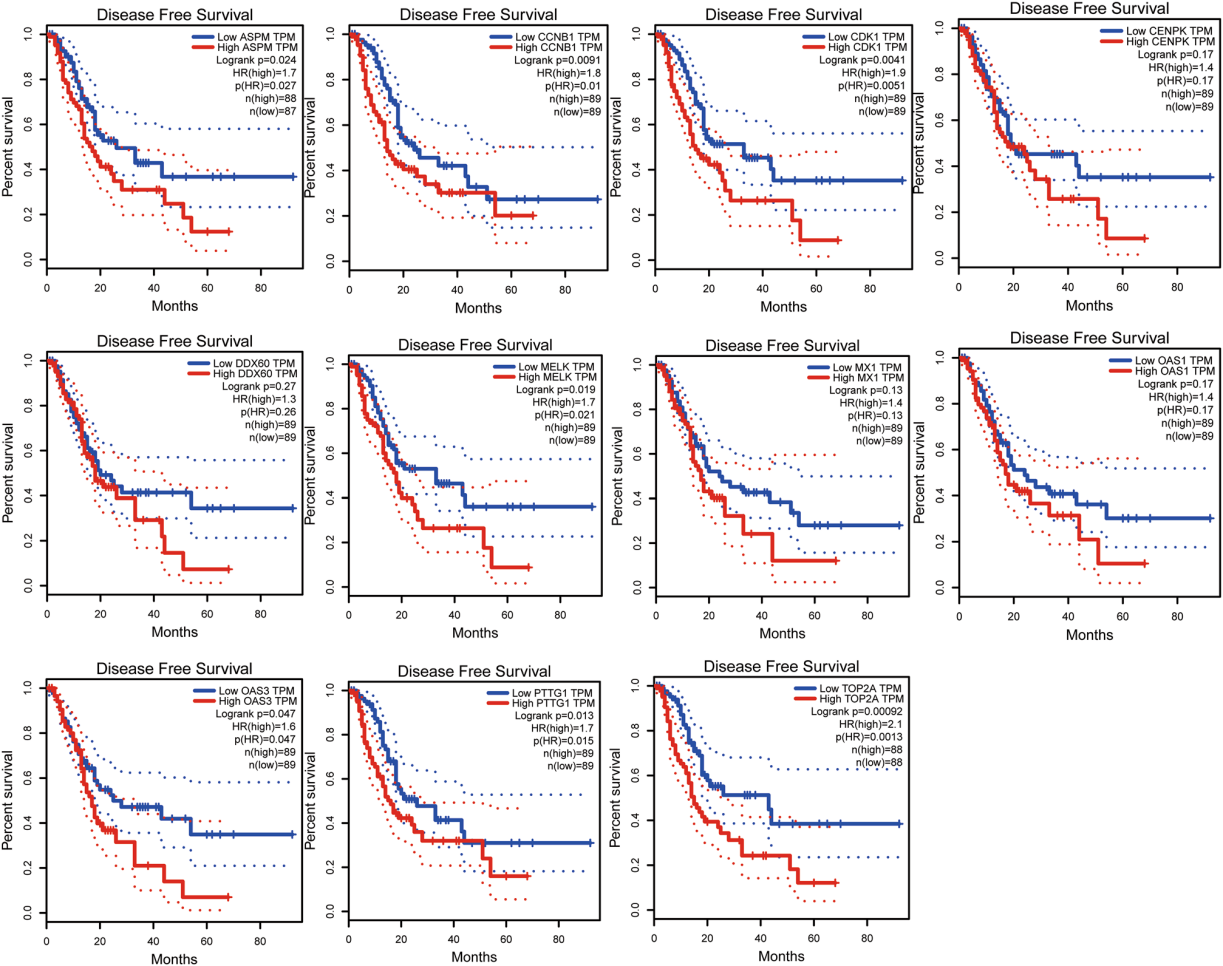
Analysis of correlation between the expression of central genes and the disease free survival of PDAC patients via GEPIA.

**Figure 7:**
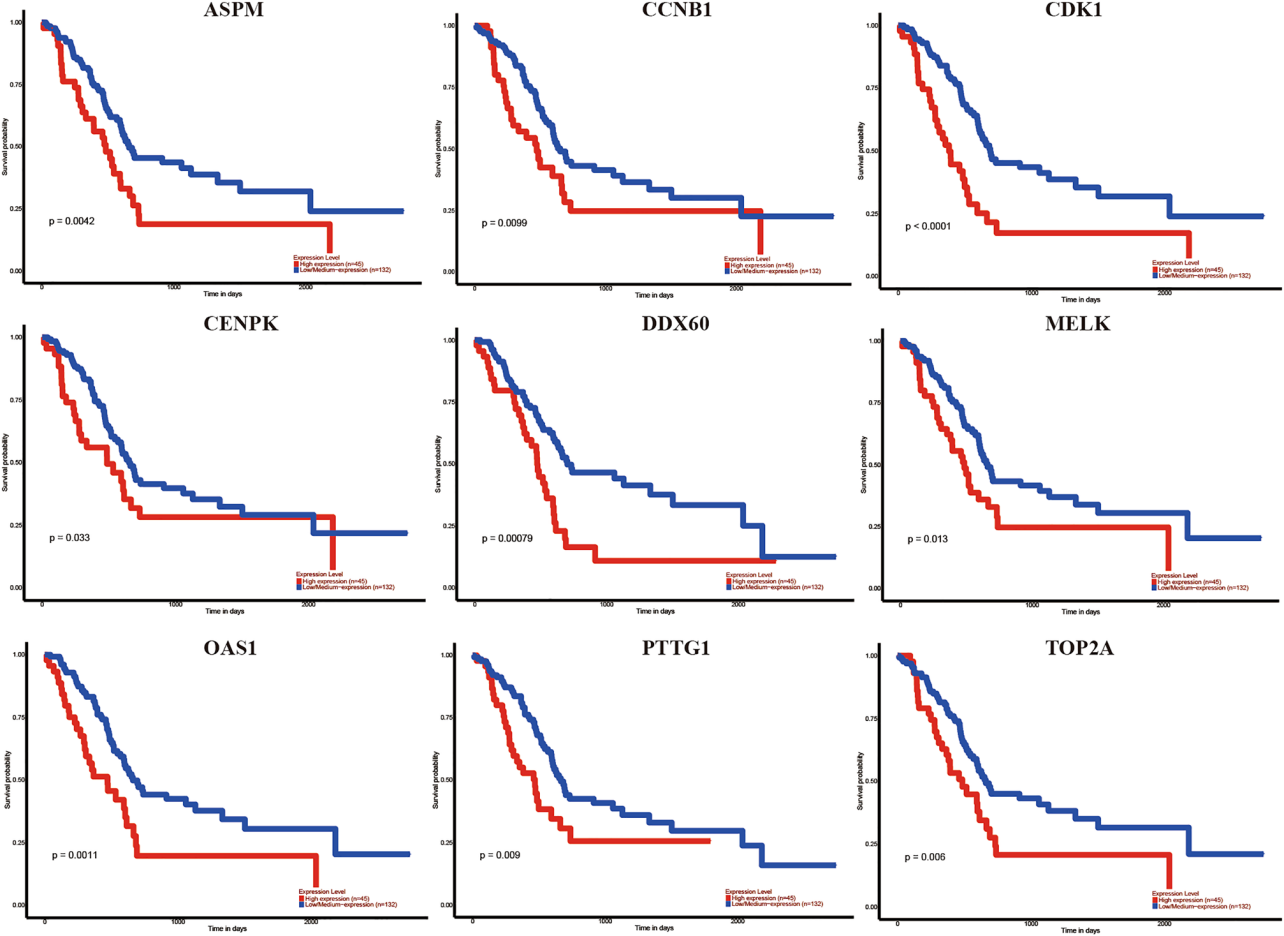
Analysis of correlation between the expression of central genes and the overall survival of PDAC patients via UALCAN.

**Figure 8:**
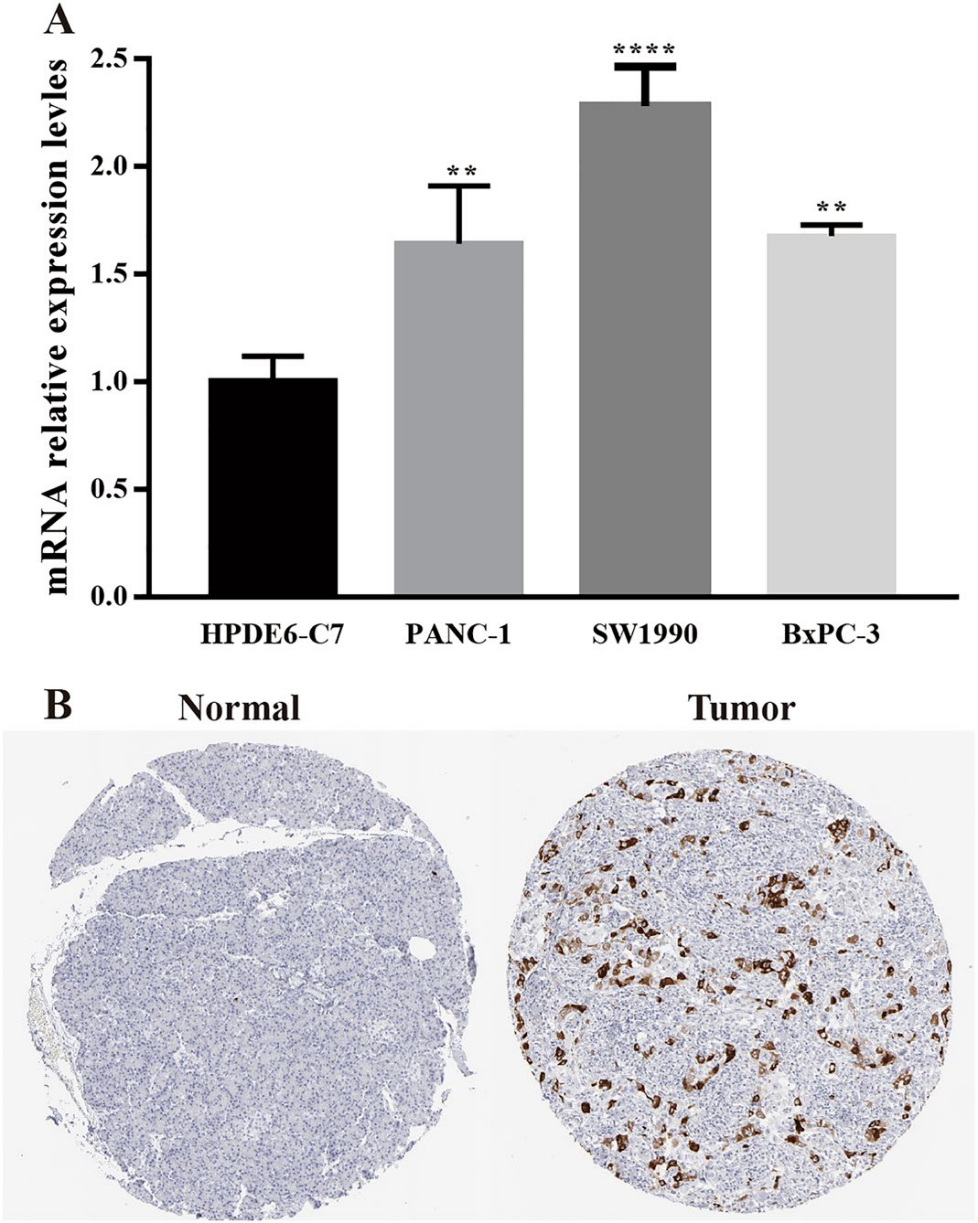
Expression validation of CCNB1 at mRNA and protein level. (**A**) Relative mRNA expression of CCNB1 in PANC-1, SW1990 and BxPC-3 compared with HPDE6-C7 cells. (**B**) The IHC staining of CCNB1 in normal and tumor tissues from HPA database was displayed. The antibody information is CCNB1 (CAB003804). * indicates *P* < 0.05, ** indicates *P* < 0.01, *** indicates *P* < 0.001, **** < indicates *P* < 0.0001.

By using GEPIA online software, we investigated the effect of 15 central genes on the overall survival of pancreatic cancer patients, and the expression levels of these genes. The data suggested that 11 highly expressed genes, namely ASPM, CCNB1, CDK1, CENPK,, DDX60, MELK, MX1, OAS1, OAS3, PTTG1 and TOP2A were thought to be remarkably associated with shorter overall survival in pancreatic cancer patients (Fig. 4), and these genes significantly higher in tumor tissues than in normal tissues (Fig. 5A–K). ASPM, CCNB1, CDK1, MELK, OAS3, PPTG1 and TOP2A were thought to be significantly associated with shorter overall survival in pancreatic cancer patients (Fig. 6). We performed the same analysis on these 15 genes using UALCAN and found that there were nine genes were significantly associated with survival of pancreatic cancer patients (Fig. 7) while only the expression of CCNB1 was significantly different between tumor tissues and normal tissues via ENCORI pan-cancer analysis (Fig. 5L). Therefore, we took CCNB1 as a candidate target gene for mRNA level detection. The qPCR assay indicated that the expression of CCNB1 was obviously increased in the PANC-1, SW1990 and BxPC-3 cells compared to the HPDE6-C7 cell (Fig. 8A). Furthermore, inmmunohistochemical results and patient clinical data were obtained from the HPA database, which showed significantly higher in situ expression of CCNB1 in pancreatic cancer tissues as compared with normal pancreatic tissues (Fig. 8B).

## Discussion

PDAC is one of the most aggressive and deadliest solid malignancies with increasing incidence and mortality in recent year^18^. Evidence suggests that PDAC will become the second leading cause of cancer death within the next 10 years^19^. PDAC patients have been reported to live an average of 4 months without any treatment. As PDAC is a highly aggressive malignant tumor and its treatment options are limited, the survival time has not been significantly prolonged due to untimely detection even with treatment^20^. Therefore, early accurate diagnosis and effective targeted therapy for pancreatic cancer are particularly important.

Previous studies had identified several biomarkers associated with pancreatic cancer. In this study, we selected four datasets from the same platform GPL570 specifically for the PDAC type of pancreatic cancer in order to ensure the uniformity and reliability of data analysis. 108 up-regulated DEGs and 7 down-regulated DEGs were screened, which significantly enriched in 8 important pathways including ECM-receptor interaction, PI3K/AKT signaling pathway, et al. Abnormal signaling pathways were important hallmarks of tumors and crucial to the occurrence and development of tumors. ECM exists in both the basement membrane and the interstitial matrix in the body. The main components of the basement membrane are collagen IV and laminin, which separate the epithelial or endothelial cell layer from the connective tissue layer^21,22^. The expression level of laminin β3 (LAMB3) were upregulated in the ECM of many tissues including pancreatic cancer, lung cancer, colon cancer^23,24^. Furtherly, Zhang et al. found that inhibition of LAMB3 counteracted the cell proliferation, invasion and migration caused by activation of PI3K/AKT signaling pathway in PDAC^24^. As a major component of the extracellular matrix, collagen plays a crucial role in the tumor microenvironment of PDAC such as cell adhesion, migration, ECM remodeling and EMT^25^. An wouding-healing experiment had shown that collagen type I is important in PDAC cell migration and metastasis^26^. During the malignant transformation from normal pancreas to PDAC, collagen type I induced MMP-2/9 activity and stimulated tumor invasion. A recent study demonstrated that serum hyaluronan and propeptide of type III were higher at baseline in PDAC patients than healthy subjects, and were associated with poor survival of PDAC patients^27^. The PI3K regulated signaling pathway network could recognize the dynamic signaling of the tumor microenvironment (TME), and could directly promote a variety of oncogenic processes or activate parallel interconnected signaling nodes^28^. More and more research had confirmed that increased activation of PI3K signaling pathway is closely related to poor overall survival in PDAC patients. Overexpression of AKT was observed in 10–20% PDAC patients, and increased AKT hyperphosphorylation levels and activity were found in approximately 60% of PDAC samples^29^. Studies had suggested that the use of PI3K inhibitors combined with small molecule attenuators of the downstream effector could effectively prevent the progression of PDAC. Studies had shown that Urolithin A could inhibit the PI3K/AKT/mTOR signaling pathway, thereby effectively reprogramming the fibroinflammatory tumor stroma, by reducing the immunosuppressive tumor-associated macrophages (TAMs) and increasing the recruitment of T cells in the TME of PDAC to achieve the effect of promoting the anti-tumor immune microenvironment^30^. Taken together, pathway enrichment analysis in this study reconfirmed the results of previous research.

Thereafter, we constructed the PPI network and screened 11 candidate genes including ASPM, CDK1, CENPK, DDX60, MELK, MX1, OAS1, OAS3, PTTG1, TOP2A and CCNB1 via STRING and Cytoscape. We performed over survival analysis on candidate genes using GEPIA and UALCAN, respectively, took the intersection, and found that a total of 9 genes were associated with the survival and prognosis of PDAC patients. The analysis via GEPIA showed that the expression levels of 11 candidate genes in PDAC tissues were higher than in normal tissues. But the data from UALCAN indicated that only the expression of CCNB1 between PDAC and normal tissues was significantly different. Subsequently, we examined the mRNA expression of CCNB1 in pancreatic cancer cell lines and the protein level via HPA database. The results showed that the expression of CCNB1 in PDAC group was higher than normal group both at the mRNA and protein level. Given the above results, we considered that CCNB1 may play important roles in PDAC tumorigenesis and progression.

As an important member of the cyclin family, CCNB1 is an important cell cycle regulator involved in the regulation of G2/M checkpoints, and can interact with cyclin-dependent kinase (CDK1) forms a complex, phosphorylate the substrates, and ensure that cells enter the G2/M phase from G1/S phase to promote mitosis^31–33^. The overexpression of CCNB1 were reported in several tumors such as renal, liver, breast^34–36^. The oncogene c-myc could activate the transcription of the m7G methyltransferase WDR4, which enhanced the translation of CCNB1 through transcriptional regulation, thereby promoting the progression of hepatocellular carcinoma^36^. 5MeOIndox could induce G2/M arrest of PDAC cells via inhibition of CDK1/CCNB1levels, thereby leading to apoptosis^37^. Analysis of 107 samples showed that CCNB1 was associated with poor prognosis in patients with pancreatic neuroendocrine tumors^38^. Many studies had showed that CCNB1 was involved in p53 signaling pathway. Silence of CCNB1 could inhibit proliferation, decrease the ratio of S-phase cell and the expression level of MDM2, induce apoptosis, senescence, and increase G0/G1-phase cell proportion, suggesting that these may be caused by the activation of p53 signaling pathway.

Silence of CCNB1 could inhibit the proliferation and promote senescence of Capan-2 cell via activation of p53 signaling pathway^39^. CCNB1 could promote the phosphorylation of PI3K and AKT in liver cancer and reduce p53 protein expression by promoting p53 ubiquitination^36^. This was consistent with the PI3K/AKT and p53 signaling pathways mentioned in above enrichment analysis.

Of course, our study has certain limitations and further exploration is needed. On one hand, only four datasets were selected for analysis, which should be validated in more samples in the future. On the other hand, some experiments such as knockdown assays should be performed to determine the mechanism of these hub genes in PDAC progression.

## Materials and methods

### Collection of data

We downloaded the gene expression profiles of PDAC and normal (or adjacent) samples from the GEO database, which is an open source database that freely stores data including expression profiles, sequencing, and more, where researchers can easily access, download, and process raw data. Four datasets including GSE15471, GSE32688, GSE46234 and GSE 46385 were selected, which contain 36 matching pairs of pancreatic tumor and adjacent non-tumor tissues, 25 pancreatic tumors and 7 normal pancreas tissues, 2 pancreatic tumors and 4 adjacent non-tumor tissues, 2 pancreatic tumors and 3 adjacent non-tumor tissues. All datasets were based on GPL570 platform and all tumor tissues were pathologically identified^40–43^as PDAC (Table 3).

**Table 3:** Information of datasets in the analysis of PDAC tissues vs. normal or adjacent tissues.

Author, year	GEO accession	Platform	Tissue types and sample numbers		
			PDAC	Adjacent	Count
Badea et al. (2008)^40^	GSE15471	GPL570	36	36	72
Donahue et al. (2012)^41^	GSE32688	GPL570	25	7	32
Tjora et al. (2013)^42^	GSE46234	GPL570	2	4	6
Newhook et al. (2013)^43^	GSE46385	GPL570	2	3	5

### Identification of DEGs

GEO2R, an online analysis tool, was applied to compare and filter DEGs present in the raw data of gene profiles. The cutoff criteria were |logFC|≥ 1 and *P* value < 0.05, which were considered statistically significant, where the logFC of up-regulated genes was ≥ 1, and the logFC of down-regulated genes was ≤ − 1. The Venn software online (http://bioinformatics.psb.ugent.be/webtools/Venn/) was used to screen and show the DEGs in the three datasets.

### Enrichment analysis of DEGs via gene ontology and Kyoto encyclopedia of genes and genomes pathway

In order to further investigate the function and relationship of DEGs, Database Annotation, Visualization, and Integrated Discovery (DAVID 2021 update, http://david.ncifcrf.gov)^44^ was applied to perform enrichment analysis covering Biological Process (BP), Cellular Component (CC), Molecular Function (MF) and KEGG pathway analysis. *P*-value < 0.05 indicated statistically significant.

### Construction of the PPI network and analysis of the module

The Search Tool for the Retrieval of Interacting Genes/Proteins (STRING, https://cn.string-db.org/cgi/input.pl)^45^ was used to build the PPI network for analyze the relationship between the differently expressed proteins and further clarify the specific relationship between genes and disease. Cytoscape (version 3.8.2)^46^ is the software that can analyze and visualize the PPI network. As a component of Cytoscape, Molecular complex detection (MCODE) was used to decipher the network protein association and identify gene clusters (highly interconnected regions); the criteria are as follows: node score cutoff = 0.2; degree cutoff = 2; k‑core = 2 and Max. Depth = 100.

### Analysis of survival and expression validation of hub genes in TGCA dataset

The Gene Expression Profiling Interactive Analysis tool (GEPIA, http://gepia.cancer-pku.cn/)^47^ is an useful resource for the analysis of the Cancer Genome Atlas (TCGA) and Genotype-tissue Expression data. The analysis of expression and interaction of normal and cancer genes, and pathological stage, prognostic analysis of genes in normal and cancer tissues were performed via GEPIA. UALCAN (http://ualcan.path.uab.edu/index.html)^48^ was applied to analyze the correlation between pancreatic cancer patients survival prognosis and hub gene expression. The Human Protein Atlas (HPA) (http://www.proteinatlas.org/) was used for validating the protein expression level of hub genes. Encyclopedia of RNA Interactomes (ENCORI)^49^ (http://starbase.sysu.edu.cn/) is an open-source bioinformatics platform to study RNA-RNA interactions, targets prediction, signaling pathways and pan-cancer differential expression and survival analysis.

### Gene expression assay at mRNA level via quantitative real-time polymerase chain reaction

We extracted total RNA from cells using EasyPure RNA Purification Kit (Tansgen, China). Purity measurement confirmed that A260/280 was between 1.8 and 2.0 and the concentration is 800-1000 ng/μl, all of which met the requirements of further experiments. The quantitative real-time PCR was examined by SYBR Green (Takara, Japan) on the LightCycler 96 Real-Time PCR Systems (Roche, Switzerland). All the primers were designed with Primer 7.0 software and the sequences of primer were listed in Table 4. GAPDH was chosen as the internal reference.

**Table 4:** Primers used for real-time PCR analysis.

Gene name	Primer sequence
CCNB1	Forward: 5′-AATAAGGCGAAGATCAACATGGC-3′ Reverse: 5′-TTTGTTACCAATGTCCCCAAGAG-3′
GAPDH	Forward: 5′-TGGGTGTGAACCATGAGAAGT-3′ Reverse: 5′-TGAGTCCTTCCACGATACCAA-3′

### Statistical analysis

Every experiment in vitro was conducted at least three times. SPSS 25.0 was used for conducting the statistical analysis. *P* values of less than 0.05 (*) were considered statistically significant.

## Conclusion

In summary, we performed an integrative bioinformatics approach to screen 115 DEGs from three PDAC GEO datasets, in which CCNB1was identified that may serve as potential diagnostic and therapeutic biomarker in PDAC patients. In the future, we will further study the molecular mechanism of the occurrence and development of pancreatic cancer through biological experiments by establishing stable gene silencing transfected cell models. These results will provide new strategies for the diagnosis, treatment and prognosis of PDAC patients.

## Supplementary Information

Supplementary Tables.

## Data Availability

The microarray data used to support the findings of this study were deposited in the Gene Expression Omnibus (GEO, https://www.ncbi.nlm.nih.gov/geo/) repository (accession numbers: GSE15471, GSE32688, GSE46234 and GSE46385).

## References

[1] Kamisawa T, Wood LD, Itoi T (2016). Pancreatic cancer. Lancet.

[2] Sung H, Ferlay J, Siegel RL (2021). Global cancer statistics 2020: GLOBOCAN estimates of incidence and mortality worldwide for 36 cancers in 185 countries. CA Cancer J. Clin..

[3] Siegel RL, Miller KD, Fuchs HE (2021). Cancer statistics, 2021. CA Cancer J. Clin..

[4] Arnold M, Rutherford MJ, Bardot A (2019). Progress in cancer survival, mortality, and incidence in seven high-income countries 1995–2014 (ICBP SURVMARK-2): A population-based study. Lancet Oncol..

[5] Ushio J, Kanno A, Ikeda E (2021). Pancreatic ductal adenocarcinoma: Epidemiology and risk factors. Diagnostics..

[6] Zhang L, Sanagapalli S, Stoita A (2018). Challenges in diagnosis of pancreatic cancer. World J. Gastroenterol..

[7] McGuire, S. World Cancer Report 2014. Geneva, Switzerland: World Health Organization, International Agency for Research on Cancer, WHO Press, 2015. *Adv. Nutr.* **7**(2), 418–419 (2016). 10.3945/an.116.012211 PMC4785485 26980827

[8] Bray F, Ferlay J, Soerjomataram I (2018). Global cancer statistics 2018: GLOBOCAN estimates of incidence and mortality worldwide for 36 cancers in 185 countries. CA Cancer J. Clin..

[9] Rawla P, Sunkara T, Gaduputi V (2019). Epidemiology of pancreatic cancer: Global trends, etiology and risk factors. World J. Oncol..

[10] Kern SE, Shi C, Hruban RH (2011). The complexity of pancreatic ductal cancers and multidimensional strategies for therapeutic targeting. J. Pathol..

[11] Grasso C, Jansen G, Giovannetti E (2017). Drug resistance in pancreatic cancer: Impact of altered energy metabolism. Crit. Rev. Oncol. Hematol..

[12] Sjoblom T, Jones S, Wood LD (2006). The consensus coding sequences of human breast and colorectal cancers. Science (New York, NY)..

[13] Wood LD, Parsons DW, Jones S (2007). The genomic landscapes of human breast and colorectal cancers. Science (New York, NY)..

[14] Bass AJ, Lawrence MS, Brace LE (2011). Genomic sequencing of colorectal adenocarcinomas identifies a recurrent VTI1A-TCF7L2 fusion. Nat. Genet..

[15] Shih W, Chetty R, Tsao MS (2005). Expression profiling by microarrays in colorectal cancer (review). Oncol. Rep..

[16] Rifai N, Gillette MA, Carr SA (2006). Protein biomarker discovery and validation: The long and uncertain path to clinical utility. Nat. Biotechnol..

[17] Segata N, Izard J, Waldron L (2011). Metagenomic biomarker discovery and explanation. Genome Biol..

[18] Siegel R, Ma J, Zou Z (2014). Cancer statistics, 2014. CA Cancer J. Clin..

[19] Li YJ, Wu JY, Wang JM (2020). Emerging nanomedicine-based strategies for preventing metastasis of pancreatic cancer. J. Control. Release.

[20] Wang X, Wang L, Mo Q (2015). Changes of Th17/Treg cell and related cytokines in pancreatic cancer patients. Int. J. Clin. Exp. Pathol..

[21] LeBleu VS, Macdonald B, Kalluri R (2007). Structure and function of basement membranes. Exp. Biol. Med..

[22] Jayadev R, Sherwood DR (2017). Basement membranes. Curr. Biol. CB..

[23] Tian C, Clauser KR, Ohlund D (2019). Proteomic analyses of ECM during pancreatic ductal adenocarcinoma progression reveal different contributions by tumor and stromal cells. Proc. Natl. Acad. Sci. U.S.A..

[24] Zhang H, Pan YZ, Cheung M (2019). LAMB3 mediates apoptotic, proliferative, invasive, and metastatic behaviors in pancreatic cancer by regulating the PI3K/Akt signaling pathway. Cell Death Dis..

[25] Huang H, Wright S, Zhang J (2019). Getting a grip on adhesion: Cadherin switching and collagen signaling. Biochim. Biophys. Acta.

[26] Procacci P, Moscheni C, Sartori P (2018). Tumor(-)stroma cross-talk in human pancreatic ductal adenocarcinoma: A focus on the effect of the extracellular matrix on tumor cell phenotype and invasive potential. Cells.

[27] Chen IM, Willumsen N, Dehlendorff C (2020). Clinical value of serum hyaluronan and propeptide of type III collagen in patients with pancreatic cancer. Int. J. Cancer.

[28] Mehra S, Deshpande N, Nagathihalli N (2021). Targeting PI3K pathway in pancreatic ductal adenocarcinoma: Rationale and progress. Cancers.

[29] Schlieman MG, Fahy BN, Ramsamooj R (2003). Incidence, mechanism and prognostic value of activated AKT in pancreas cancer. Br. J. Cancer.

[30] Totiger TM, Srinivasan S, Jala VR (2019). Urolithin A, a novel natural compound to target PI3K/AKT/mTOR pathway in pancreatic cancer. Mol. Cancer Ther..

[31] Nam HJ, van Deursen JM (2014). Cyclin B2 and p53 control proper timing of centrosome separation. Nat. Cell Biol..

[32] Wang Z, Fan M, Candas D (2014). Cyclin B1/Cdk1 coordinates mitochondrial respiration for cell-cycle G2/M progression. Dev. Cell.

[33] Chiu HC, Huang WR, Liao TL (2018). Mechanistic insights into avian reovirus p17-modulated suppression of cell cycle CDK-cyclin complexes and enhancement of p53 and cyclin H interaction. J. Biol. Chem..

[34] Ikuerowo SO, Kuczyk MA, Mengel M (2006). Alteration of subcellular and cellular expression patterns of cyclin B1 in renal cell carcinoma is significantly related to clinical progression and survival of patients. Int. J. Cancer.

[35] Ding K, Li W, Zou Z (2014). CCNB1 is a prognostic biomarker for ER+ breast cancer. Med. Hypotheses.

[36] Xia P, Zhang H, Xu K (2021). MYC-targeted WDR4 promotes proliferation, metastasis, and sorafenib resistance by inducing CCNB1 translation in hepatocellular carcinoma. Cell Death Dis..

[37] Sano M, Ichimaru Y, Kurita M (2017). Induction of cell death in pancreatic ductal adenocarcinoma by indirubin 3'-oxime and 5-methoxyindirubin 3'-oxime in vitro and in vivo. Cancer Lett..

[38] Shin JU, Lee CH, Lee KT (2012). Prognostic significance of ATM and cyclin B1 in pancreatic neuroendocrine tumor. Tumour Biol..

[39] Zhang H, Zhang X, Li X (2018). Effect of CCNB1 silencing on cell cycle, senescence, and apoptosis through the p53 signaling pathway in pancreatic cancer. J. Cell. Physiol..

[40] Badea L, Herlea V, Dima SO (2008). Combined gene expression analysis of whole-tissue and microdissected pancreatic ductal adenocarcinoma identifies genes specifically overexpressed in tumor epithelia. Hepatogastroenterology.

[41] Donahue TR, Tran LM, Hill R (2012). Integrative survival-based molecular profiling of human pancreatic cancer. Clin. Cancer Res..

[42] Tjora E, Wathle G, Engjom T (2013). Severe pancreatic dysfunction but compensated nutritional status in monogenic pancreatic disease caused by carboxyl-ester lipase mutations. Pancreas.

[43] Newhook TE, Blais EM, Lindberg JM (2014). A thirteen-gene expression signature predicts survival of patients with pancreatic cancer and identifies new genes of interest. PLoS ONE.

[44] Dennis G, Sherman BT, Hosack DA (2003). DAVID: Database for annotation, visualization, and integrated discovery. Genome Biol..

[45] Szklarczyk D, Morris JH, Cook H (2017). The STRING database in 2017: Quality-controlled protein–protein association networks, made broadly accessible. Nucleic Acids Res..

[46] Kohl M, Wiese S, Warscheid B (2011). Cytoscape: Software for visualization and analysis of biological networks. Methods Mol. Biol..

[47] Tang Z, Li C, Kang B (2017). GEPIA: A web server for cancer and normal gene expression profiling and interactive analyses. Nucleic Acids Res..

[48] Chandrashekar DS, Bashel B, Balasubramanya SAH (2017). UALCAN: A portal for facilitating tumor subgroup gene expression and survival analyses. Neoplasia (New York, NY)..

[49] Li JH, Liu S, Zhou H (2014). starBase v2.0: Decoding miRNA-ceRNA, miRNA-ncRNA and protein-RNA interaction networks from large-scale CLIP-Seq data. Nucleic Acids Res..

