# Supplementary material for: Identification and verification of CCNB1 as a potential prognostic biomarker by comprehensive analysis
Source: Sci Rep. 2022 Sep 27;12:16153. doi: 10.1038/s41598-022-20615-8 (PMC9515086; doi:10.1038/s41598-022-20615-8)
Supplement: Supplementary file 1 — Supplementary Tables. [file 41598_2022_20615_MOESM1_ESM.pdf]

Supplementary table 1. Terms of BP were shown by enrichment analysis ($P < 0.05$)

Term	Count	P-Value	Genes
GO:0016477~cell migration	12	2.94E-07	EFNA1, DOCK5, CEACAM1, JUP, SDC4, COL5A1, LAMB3, ITGB4, CDK1, ADAM9, SDC1, CTHRC1
GO:0007229~integrin-mediated signaling pathway	8	2.67E-06	FERMT1, COL3A1, CEACAM1, ITGB4, ITGA2, ADAM9, ADAM10, ISG15
GO:0045071~negative regulation of viral genome replication	6	5.51E-06	OAS1, SLPI, OAS3, MX1, ISG15, IFIT1
GO:0042060~wound healing	7	2.36E-05	COL1A1, COL3A1, POSTN, SDC4, SDC1, EPPK1, DCBLD2
GO:0043589~skin morphogenesis	4	2.66E-05	COL1A1, COL1A2, ITGB4, ITGA2
GO:0006915~apoptotic process	14	3.20E-05	EGLN3, BIK, IGFBP3, MX1, TEX11, IFI6, PYCARD, MELK, CEACAM6, CEACAM5, CDK1, LCN2, IAPP, TNFRSF21
GO:0009615~response to virus	7	3.61E-05	OAS1, OAS3, MX1, ISG15, DDX60, IFIT1, DUOX2
GO:0051607~defense response to virus	9	4.47E-05	PYCARD, RTP4, OAS1, OAS3, MX1, IFI6, ISG15, DDX60, IFIT1
GO:0007155~cell adhesion	13	6.21E-05	LGALS3BP, POSTN, JUP, ITGB4, PCDH7, ITGA2, LAMC2, COL1A1, FERMT1, CLDN4, CEACAM1, COL5A1, ADAM9
GO:0030198~extracellular matrix organization	7	3.20E-04	ERO1A, COL1A1, COL3A1, POSTN, COL1A2, COL5A1, ELF3
GO:0030199~collagen fibril organization	5	3.46E-04	COL1A1, COL3A1, COL1A2, COL5A1, ANXA2
GO:0009612~response to mechanical stimulus	5	4.17E-04	COL1A1, COL3A1, POSTN, BTG2, CCNB1
GO:0010756~positive	3	8.38E-04	ANXA2, MELTF, S100A10

regulation of plasminogen activation			
GO:0043588~skin development	4	0.001222	COL1A1, COL3A1, JUP, COL5A1
GO:0042493~response to drug	8	0.001254	COL1A1, NQO1, CCNB1, ITGA2, CDK1, LCN2, INHBA, S100A10
GO:0045087~innate immune response	11	0.001805	PYCARD, LGALS3, OAS1, SLPI, OAS3, MX1, IFI6, LCN2, PI3, DDX60, CD55
GO:0030855~epithelial cell differentiation	5	0.001872	LGALS3, KRT19, FZD2, ELF3, CDK1
GO:0048333~mesodermal cell differentiation	3	0.001947	ITGB4, ITGA2, INHBA
GO:0001568~blood vessel development	4	0.001998	COL1A1, CEACAM1, COL1A2, COL5A1
GO:0042552~myelination	4	0.002862	ADGRG6, MALL, MAL2, TNFRSF21
GO:0007160~cell-matrix adhesion	5	0.002999	FERMT1, COL3A1, ITGB4, ITGA2, ADAM9
GO:0014075~response to amine	3	0.003064	NQO1, ITGA2, CDK1
GO:0098609~cell-cell adhesion	6	0.004189	BAIAP2L1, JUP, ANXA2, PERP, ITGA2, S100A11
GO:0006979~response to oxidative stress	5	0.004519	ERO1A, NQO1, GPX2, GPX8, DUOX2
GO:2000811~negative regulation of anoikis	3	0.004918	CEACAM6, CEACAM5, PDK4
GO:0060339~negative regulation of type I interferon-mediated signaling pathway	3	0.005445	OAS1, OAS3, ISG15
GO:0014911~positive regulation of smooth muscle cell migration	3	0.007795	POSTN, ITGA2, S100A11
GO:0043616~keratinocyte proliferation	3	0.007795	SDR16C5, FERMT1, SFN

GO:0071356~cellular response to tumor necrosis factor	5	0.008341	PYCARD, COL1A1, POSTN, LCN2, TNFRSF21
GO:0098869~cellular oxidant detoxification	4	0.00906	GPX2, ALB, GPX8, DUOX2
GO:0001954~positive regulation of cell-matrix adhesion	3	0.010524	FERMT1, JUP, DMD
GO:0030335~positive regulation of cell migration	6	0.014172	COL1A1, CLDN4, CEACAM6, ADAM9, ADAM10, LAMC2
GO:0042476~odontogenesis	3	0.015285	COL1A2, SDC1, INHBA
GO:0009888~tissue development	3	0.016153	POSTN, LAMB3, LAMC2
GO:1905686~positive regulation of plasma membrane repair	2	0.016579	ANXA2, S100A10
GO:0033627~cell adhesion mediated by integrin	3	0.020799	ITGB4, ITGA2, ADAM9
GO:0071659~negative regulation of IP-10 production	2	0.022044	OAS1, OAS3
GO:1905448~positive regulation of mitochondrial ATP synthesis coupled electron transport	2	0.022044	CCNB1, CDK1
GO:1903553~positive regulation of extracellular exosome assembly	2	0.022044	SDC4, SDC1
GO:0032728~positive regulation of interferon-beta production	3	0.022795	OAS1, OAS3, ISG15
GO:0008584~male gonad development	4	0.024567	ASPM, BIK, TEX11, INHBA
GO:0060700~regulation of ribonuclease activity	2	0.027479	OAS1, OAS3
GO:0051591~response to	3	0.029229	COL1A1, SDC1, DUOX2

cAMP			
GO:2000342~negative regulation of chemokine (C-X-C motif) ligand 2 production	2	0.032885	OAS1, OAS3
GO:0001765~membrane raft assembly	2	0.032885	ANXA2, S100A10
GO:0031346~positive regulation of cell projection organization	2	0.032885	ITGA2, LCN2
GO:0097327~response to antineoplastic agent	2	0.032885	ADAM9, ADAM10
GO:0030838~positive regulation of actin filament polymerization	3	0.035076	PYCARD, BAIAP2L1, CDC42EP5
GO:0042542~response to hydrogen peroxide	3	0.035076	COL1A1, ADAM9, SDC1
GO:0043065~positive regulation of apoptotic process	6	0.036234	TOP2A, PYCARD, MELK, IGFBP3, ADAM10, IAPP
GO:0070106~interleukin-27-mediated signaling pathway	2	0.03826	OAS1, MX1
GO:0043434~response to peptide hormone	3	0.038783	COL1A1, BTG2, TFF1
GO:0048146~positive regulation of fibroblast proliferation	3	0.040051	CCNB1, ANXA2, S100A6
GO:0071230~cellular response to amino acid stimulus	3	0.042633	COL1A1, COL3A1, COL1A2
GO:0000086~G2/M transition of mitotic cell cycle	3	0.043946	CCNB1, MELK, CDK1
GO:0010951~negative regulation of endopeptidase activity	4	0.047911	PTTG1, ANXA2, SLPI, PI3
GO:0055015~ventricular cardiac muscle cell	2	0.048923	CCNB1, CDK1

development

Supplementary table 2. Terms of CC were shown by enrichment analysis (*P* < 0.05)

Term	Count	PValue	Genes
GO:0070062~extracellular exosome	40	4.63E-12	LGALS3BP, BTG2, SLC44A4, SDC4, MTMR11, ITGB4, LY75, CAPG, LGALS3, BAIAP2L1, MAL2, GCNT3, SFN, TSPAN1, CD55, DUOX2, S100A11, S100A10, SLC12A2, MARCKSL1, AOC1, JUP, ANXA2, ADAM10, TMC5, KRT19, CEACAM1, GPRC5A, COL1A2, SLPI, FXYD3, CEACAM5, ALB, S100A6, CDK1, LCN2, SDC1, ADAM9, S100P, MELTF
GO:0005576~extracellular region	33	3.37E-08	LGALS3BP, LAMC2, PYCARD, LGALS3, AGR2, PI3, CD55, S100A11, CTHRC1, S100A10, AOC1, LAMB3, JUP, ANXA2, IGFBP3, ISG15, INHBA, EFNA1, COL1A1, COL3A1, COL1A2, COL5A1, OAS1, SLPI, CEACAM5, ALB, S100A6, LCN2, S100P, IAPP, TFF1, MELTF, MUC20
GO:0009986~cell surface	17	2.66E-07	ANXA2, SDC4, ITGB4, ITGA2, ADAM10, DCBLD2, LGALS3, CEACAM1, CEACAM6, CEACAM5, ADGRG6, SDC1, ADAM9, DMD, MELTF, CD55, DUOX2
GO:0005615~extracellular space	29	7.03E-07	ERO1A, LGALS3BP, LAMC2, CAPG, LGALS3, AGR2, SFN, PI3, S100A11, CTHRC1, S100A10, POSTN, AOC1, ANXA2, IGFBP3, INHBA, COL1A1, COL3A1, COL1A2, COL5A1, SLPI, CEACAM6, OAS3, ALB, LCN2, ADAM9, IAPP, TFF1, MELTF
GO:0005886~plasma	50	3.26E-06	DOCK5, SLC44A4, ITGB4, HK2,

membrane			SDR16C5, LGALS3, BAIAP2L1, PMEPA1, PAQR8, ANXA2, ITGA2, ADAM10, ACSL5, CLDN4, CEACAM1, GPRC5A, MELK, CEACAM6, CDC42EP5, CEACAM5, OAS3, MALL, S100A6, TNFRSF21, SDC4, IFI6, PERP, ADGRG6, DMD, C19ORF33, TSPAN1, CD55, DUOX2, S100A10, SLC12A2, AOC1, MARCKSL1, FZD2, JUP, OSBPL3, PCDH7, MX1, AHNAK2, EFNA1, KRT19, FXYD3, SDC1, MELTF, KCNK1, MUC20
GO:0016324~apical plasma membrane	11	3.15E-05	SLC12A2, ASPM, CLDN4, CEACAM1, SLC44A4, CEACAM6, CEACAM5, MAL2, KCNK1, MUC20, DUOX2
GO:0005788~endoplasmic reticulum lumen	10	3.82E-05	ERO1A, COL1A1, COL3A1, COL1A2, COL5A1, IGFBP3, ALB, GPX8, ADAM10, MELTF
GO:0009925~basal plasma membrane	5	1.57E-04	SLC12A2, CLDN4, CEACAM1, ITGB4, MUC20
GO:0016327~apicolateral plasma membrane	4	2.41E-04	CLDN4, KRT19, JUP, EPPK1
GO:0030054~cell junction	8	2.45E-04	FERMT1, CEACAM1, ITGB4, CAPG, EPPK1, DMD, TSPAN1, DUOX2
GO:0016328~lateral plasma membrane	5	6.24E-04	SLC12A2, CLDN4, CEACAM1, JUP, DMD
GO:0005581~collagen trimer	5	0.001483	COL1A1, COL3A1, COL1A2, COL5A1, CTHRC1
GO:0016020~membrane	26	0.00164	ERO1A, LGALS3BP, SLC44A4, MTMR11, LAMC2, HK2, LGALS3, CCNB1, GCNT3, TSPAN1, S100A10, SLC12A2, ANXA2, ITGA2, OSBPL3, MX1, ADAM10, ACSL5, CEACAM1, MELK, OAS1, CDC42EP5, OAS3, CDK1, ADAM9, EPPK1

GO:0045121~membrane raft	7	0.002264	SDC4, ANXA2, MALL, MAL2, DMD, CD55, S100A10
GO:0005912~adherens junction	6	0.002461	CEACAM1, BAIAP2L1, JUP, ANXA2, ADAM10, S100A11
GO:0071944~cell periphery	4	0.00299	SLC12A2, FERMT1, KRT19, EPPK1
GO:0005737~cytoplasm	44	0.003618	RTP4, TOP2A, DOCK5, BTG2, MTMR11, CAPG, IFIT1, DDX60, PYCARD, LGALS3, CCNB1, PTTG1, ADGRG6, DMD, SFN, TSPAN1, S100A11, CTHRC1, S100A10, NQO1, POSTN, EGLN3, MARCKSL1, GPX2, FZD2, JUP, ANXA2, MX1, AHNAK2, ADAM10, ISG15, COL1A1, ASPM, FERMT1, MELK, OAS1, CDC42EP5, OAS3, ALB, S100A6, CDK1, S100P, EPPK1, TNFRSF21
GO:0043034~costamere	3	0.004065	KRT19, SDC4, DMD
GO:0035580~specific granule lumen	4	0.004421	AOC1, JUP, SLPI, LCN2
GO:0005887~integral component of plasma membrane	17	0.006107	SLC12A2, SDC4, PCDH7, LY75, TMC5, DCBLD2, CLDN4, CEACAM1, GPRC5A, FXYD3, PERP, CEACAM5, ADGRG6, SDC1, KCNK1, TSPAN1, TNFRSF21
GO:0005925~focal adhesion	8	0.007601	FERMT1, FZD2, JUP, SDC4, ITGB4, ITGA2, ADAM9, ADAM10
GO:0016323~basolateral plasma membrane	6	0.008945	SLC12A2, ANXA2, CEACAM5, ADAM9, EPPK1, MUC20
GO:0031528~microvillus membrane	3	0.010384	CEACAM1, S100P, MUC20
GO:0005584~collagen type I trimer	2	0.010621	COL1A1, COL1A2
GO:0097125~cyclin B1-CDK1 complex	2	0.010621	CCNB1, CDK1
GO:1990665~AnxA2-p11	2	0.010621	ANXA2, S100A10

complex			
GO:0031012~extracellular matrix	6	0.012557	COL1A1, COL3A1, POSTN, COL1A2, COL5A1, PI3
GO:0005604~basement membrane	4	0.013863	COL5A1, ANXA2, ITGB4, LAMC2
GO:0001726~ruffle	4	0.016782	ANXA2, S100A6, CAPG, S100A11
GO:0005796~Golgi lumen	4	0.019062	ERO1A, SDC4, SDC1, MUC20
GO:0042383~sarcolemma	4	0.019062	KRT19, ANXA2, AHNAK2, DMD
GO:0031225~anchored component of membrane	4	0.021505	EFNA1, CEACAM6, CEACAM5, CD55
GO:0005856~cytoskeleton	8	0.025643	FERMT1, MARCKSL1, KRT19, BAIAP2L1, JUP, CDC42EP5, EPPK1, DMD
GO:0030056~hemidesmosome	2	0.036686	ITGB4, EPPK1
GO:0001533~cornified envelope	3	0.03948	JUP, ANXA2, PI3
GO:0005783~endoplasmic reticulum	12	0.040779	ERO1A, PYCARD, SDR16C5, ERO1B, COL1A2, OAS1, TMPRSS3, ALB, AGR2, ACSL5, DUOX2, S100A10
GO:0030055~cell-substrate junction	2	0.041816	FERMT1, DMD

Supplementary table 3. Terms of MF were shown by enrichment analysis ($P < 0.05$)

Term	Count	PValue	Genes
GO:0005178~integrin binding	9	4.12E-06	FERMT1, COL3A1, COL5A1, ITGB4, ITGA2, ADAM9, ADAM10, ISG15, DMD
GO:0005201~extracellular matrix structural constituent	8	1.48E-05	COL1A1, COL3A1, POSTN, COL1A2, COL5A1, LAMB3, LAMC2, CTHRC1

GO:0048407~platelet-derived growth factor binding	4	2.94E-05	COL1A1, COL3A1, COL1A2, COL5A1
GO:0002020~protease binding	6	4.13E-04	PYCARD, COL1A1, COL3A1, COL1A2, ANXA2, LCN2
GO:0043236~laminin binding	4	4.35E-04	LGALS3, ITGA2, ADGRG6, ADAM9
GO:0005515~protein binding	88	1.00E-03	TMEM45B, ERO1A, RTP4, TOP2A, LGALS3BP, DOCK5, ERO1B, BTG2, ITGB4, IFIT1, HK2, PYCARD, SDR16C5, LGALS3, BAIAP2L1, PTTG1, PDK4, PMEPA1, PAQR8, POSTN, ANXA2, IGFBP3, ITGA2, TEX11, GPX8, ADAM10, ACSL5, C15ORF48, CLDN4, CEACAM1, GPRC5A, MELK, SLPI, OAS1, ELF3, CEACAM6, CEACAM5, OAS3, MALL, S100A6, ADAM9, IAPP, TFF1, TNFRSF21, SDC4, IFI6, CAPG, DDX60, CCNB1, PERP, AGR2, MAL2, DMD, SFN, TSPAN1, S100A11, CD55, DUOX2, S100A10, SLC12A2, NQO1, EGLN3, AOC1, MARCKSL1, FZD2, JUP, LAMB3, BIK, OSBPL3, MX1, AHNAK2, ISG15, INHBA, DCBLD2, EFNA1, COL1A1, COL3A1, KRT19, COL1A2, COL5A1, ALB, CENPK, CDK1, LCN2, SDC1, S100P, MELTF, KCNK1
GO:0042802~identical protein binding	21	0.001637	NQO1, ANXA2, SDC4, MX1, INHBA, PYCARD, COL1A1, CLDN4, CEACAM1, COL1A2, CEACAM6, CEACAM5, ALB, AGR2, LCN2, SDC1, IAPP, SFN, CTSE, KCNK1, MUC20

GO:0030020~extracellular matrix structural constituent conferring tensile strength	4	0.001674	COL1A1, COL3A1, COL1A2, COL5A1
GO:0048306~calcium-dependent protein binding	5	0.001948	ANXA2, S100A6, S100P, S100A11, S100A10
GO:0044548~S100 protein binding	3	0.002841	ANXA2, S100A6, S100A11
GO:0098641~cadherin binding involved in cell-cell adhesion	3	0.004706	BAIAP2L1, ANXA2, S100A11
GO:0042803~protein homodimerization activity	11	0.007909	TOP2A, PYCARD, AOC1, CEACAM1, JUP, CEACAM5, S100A6, ADAM10, S100P, S100A11, S100A10
GO:1903981~enterobactin binding	2	0.01145	ALB, LCN2
GO:0004601~peroxidase activity	3	0.01812	GPX2, GPX8, DUOX2
GO:1902945~metalloendopeptidase activity involved in amyloid precursor protein catabolic process	2	0.02277	ADAM9, ADAM10
GO:0001730~2'-5'-oligoadenylate synthetase activity	2	0.02277	OAS1, OAS3
GO:0016972~thiol oxidase activity	2	0.02277	ERO1A, ERO1B
GO:0005198~structural molecule activity	5	0.022778	CLDN4, KRT19, JUP, LAMB3, EPPK1
GO:0005509~calcium ion binding	10	0.029039	AOC1, MELK, ANXA2, PCDH7, S100A6, CAPG, S100P, DUOX2, S100A11, S100A10
GO:0044877~macromolecular complex binding	7	0.032165	KRT19, AOC1, JUP, LAMB3, ITGA2, CAPG, INHBA
GO:0005080~protein kinase C binding	3	0.042481	TOP2A, SDC4, ADAM9

Supplementary table 4. Terms of CC were shown by enrichment analysis ($P < 0.05$)

Term	Count	PValue	Genes
hsa04512:ECM-receptor interaction	8	3.64E-06	COL1A1, COL1A2, SDC4, LAMB3, ITGB4, ITGA2, SDC1, LAMC2
hsa04115:p53 signaling pathway	5	0.002097	CCNB1, PERP, IGFBP3, CDK1, SFN
hsa05165:Human papillomavirus infection	9	0.003044	COL1A1, FZD2, COL1A2, LAMB3, ITGB4, ITGA2, MX1, ISG15, LAMC2
hsa05146:Amoebiasis	5	0.006974	COL1A1, COL3A1, COL1A2, LAMB3, LAMC2
hsa04510:Focal adhesion	6	0.016857	COL1A1, COL1A2, LAMB3, ITGB4, ITGA2, LAMC2
hsa05205:Proteoglycans in cancer	6	0.018205	COL1A1, FZD2, COL1A2, SDC4, ITGA2, SDC1
hsa04918:Thyroid hormone synthesis	4	0.01836	GPX2, ALB, GPX8, DUOX2
hsa05412:Arrhythmogenic right ventricular cardiomyopathy	4	0.019682	JUP, ITGB4, ITGA2, DMD
hsa05160:Hepatitis C	5	0.029542	CLDN4, OAS1, OAS3, MX1, IFIT1
hsa04974:Protein digestion and absorption	4	0.041548	COL1A1, COL3A1, COL1A2, COL5A1
hsa04151:PI3K-Akt signaling pathway	7	0.047964	EFNA1, COL1A1, COL1A2, LAMB3, ITGB4, ITGA2, LAMC2
